# Evaluation of the safety of using propofol for paediatric procedural sedation: A systematic review and meta-analysis

**DOI:** 10.1038/s41598-019-48724-x

**Published:** 2019-08-22

**Authors:** Sunhee Kim, Seokyung Hahn, Myoung-jin Jang, Yunhee Choi, Hyunsook Hong, Ji-Hyun Lee, Hee-Soo Kim

**Affiliations:** 1 Interdisciplinary Program in Medical Informatics, Seoul National University College of Medicine, Seoul, 03080 Korea; 2 Department of Medicine, Seoul National University College of Medicine, Seoul, 03080 Korea; 3 Division of Medical Statistics, Medical Research Collaborating Center, Seoul National University Hospital, Seoul, 03080 Korea; 4 Department of Anesthesiology and Pain Medicine, Seoul National University Hospital, Seoul, 03080 Korea

**Keywords:** Outcomes research, Paediatric research

## Abstract

Propofol is one of the most widely used drugs for paediatric procedural sedation owing to its known advantages, but some concerns remain regarding respiratory and/or cardiac complications in patients receiving propofol. Although a considerable number of randomised controlled clinical trials (RCTs) have been conducted to compare it with other sedative agents or opioids for children undergoing various procedures, propofol is still being used off-label for this indication in many countries. We performed a systematic review and meta-analysis of those RCTs to provide an overall summation of evidence that can potentially be considered for further regulatory decisions, including reimbursement policies. We searched for RCTs in MEDLINE, Embase, and the Cochrane Central Register of Controlled Trials from their inception to January 31, 2018. Our meta-analysis of 30 RCTs confirmed that propofol sedation had advantages in recovery time when compared with other drugs, without excessive concerns for cardiovascular or respiratory adverse events. Its safety profile regarding coughing, nausea or vomiting, and emergence delirium was also similar to that of other drugs. The overall evidence suggests that propofol sedation for paediatric procedures should be considered more positively in the context of regulatory decisions.

## Introduction

Propofol is an intravenously administered sedative-hypnotic agent with advantages including rapid onset and offset of action, nausea, and emergence delirium^1–3^. Due to its known advantages, propofol is commonly used to relieve anxiety and to sedate children who undergo therapeutic or diagnostic procedures such as cardiac catheterisation, endotracheal intubation, emergency orthopaedic procedures, dental procedures, and radiological imaging. For children, it is also known that propofol has a strong sedative effect that could be categorised as deep sedation or general anaesthesia^4^. Therefore, if the respiratory and cardiovascular instability that can be caused by propofol is not managed well, propofol sedation could have a somewhat higher likelihood of causing respiratory or cardiovascular adverse events than other sedative drugs. Several studies have been conducted in various settings since 2000 to evaluate the efficacy and safety of propofol in comparison with other alternative sedatives^5–8^. However, propofol is still being used off-label for paediatric procedural sedation in many countries without official approval for this extended indication in children, although it has been approved for paediatric use in general anaesthesia for certain age groups in some countries, including the US and the European Union^1,9–11^.

We performed a systematic review and meta-analysis of randomised controlled clinical trials (RCTs) comparing propofol with other sedative agents or opioids for children undergoing various procedures, to provide an overall summation of evidence that can potentially be considered for further regulatory decisions, including reimbursement policies.

## Results

### Study characteristics

Thirty studies met the inclusion criteria (see Supplementary Fig. S1)^12–41^. Although in 2 studies^19,28^, a small proportion of young adult patients was included, we did not exclude those studies from our analysis since we determined that the proportion of participants over 19 years was less than 5% based on the reported age distribution with the mean and the standard deviation in each study. Supplementary Table S1 shows the characteristics of the included studies.

The types of procedures performed in the included studies were radiological procedures involving magnetic resonance imaging (MRI) (6 studies), cardiology procedures (6 studies), dental procedures (4 studies), gastrointestinal procedures (4 studies), intubation (2 studies), orthopaedic procedures (4 studies), and other procedures (4 studies). Propofol was used alone in most of the studies for MRI, while it was also frequently used in combination with ketamine, midazolam, or opioids for other procedures. Dexmedetomidine, ketamine, or midazolam were compared with propofol sedation most frequently as a sole agent, in combination, or jointly with other drugs such as opioids.

The providers who were responsible for the administration of sedation were described as anaesthesiologists (10 studies), sedating physicians or nurses (2 studies), physicians (4 studies), and intensivists (1 study), but other studies did not specify the providers (13 studies).

### Risk of bias in the included studies

Twelve of the 30 studies (40%) used adequate randomisation methods, such as computer random number generation (Supplementary Fig. S2). Three studies were rated as ‘inadequate,’ because coin flipping^25^ or allocation by the enrolment day^39^ or admission day^19^ was used. Eleven studies (37%) reported adequate allocation concealment methods, such as central allocation or sealed envelopes. Two studies^19,39^ in which a quasi-randomisation method was used were considered inadequate for the concealment of allocation. Twelve studies (40%) reported the blinding or used only objective outcomes. Seven studies did not use a method of blinding^19,22,23,30,34,39^ or used some subjective outcomes without blinding^27^.

Twenty-five studies (83%) employed the intention-to-treat approach or reported dropout rates of less than 5%. Three studies were unclear about the completeness of data. In 8 studies^15,19,25,27,33,34,37,40^, some results were missing for outcomes mentioned in the methods section, and therefore we determined that a risk of selective reporting bias was present. Five studies reporting results from a small preliminary study^13,29^ and/or a sample size of fewer than 25 children^27,29,31,35^ were considered to have a potential risk for exposure to other sources of bias.

### Recovery time

**Figure 1:**
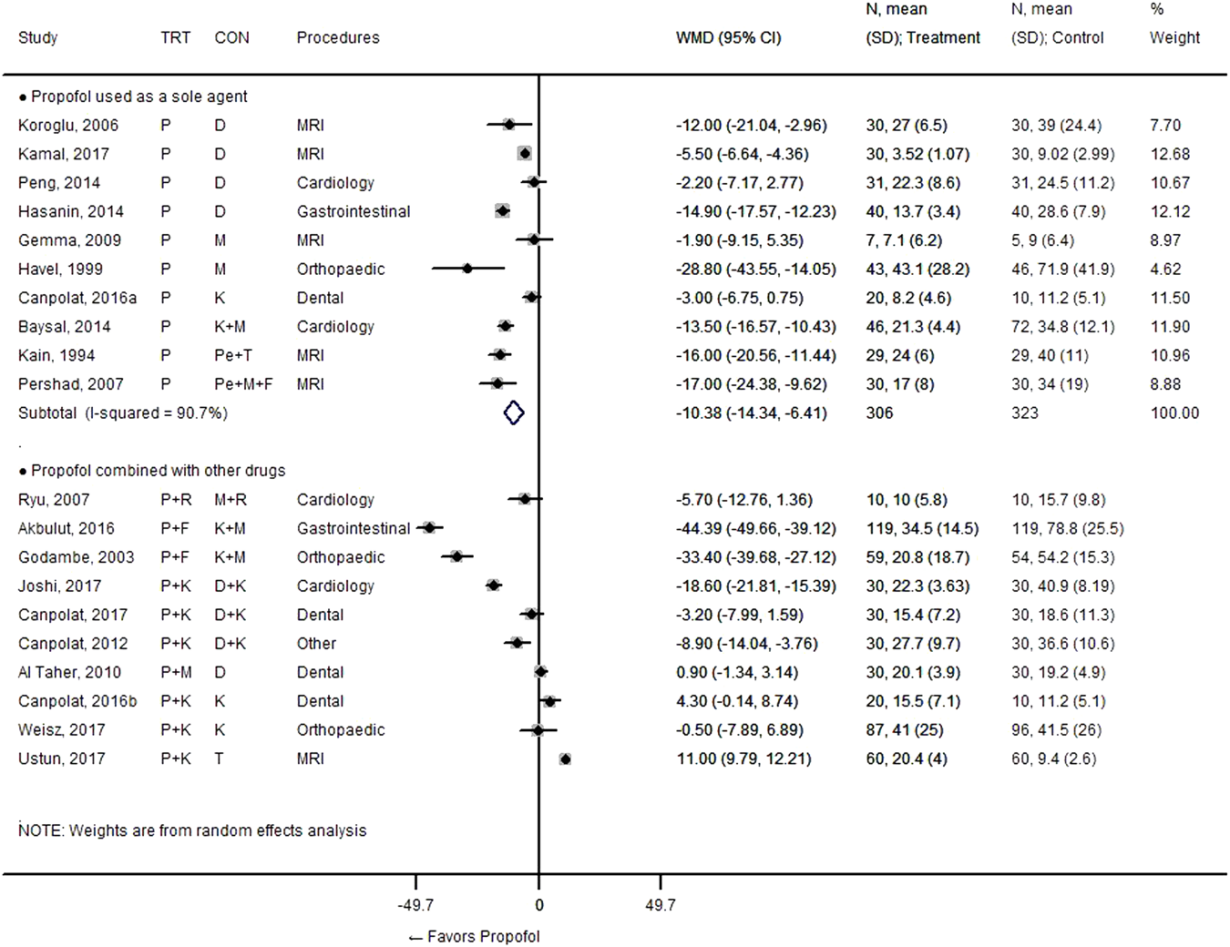
Forest plot for recovery time. Abbreviations: CI, confidence interval; CON, control; D, dexmedetomidine; F, fentanyl; K, ketamine; M, midazolam; MRI, magnetic resonance imaging; P, propofol; Pe, pentobarbital; R, remifentanil; SD, standard deviation; T, thiopental; TRT, treatment; WMD, weighted mean difference (in minutes).

Nineteen studies with 20 comparisons provided information on recovery time (Fig. 1), of which 10 evaluated propofol as a sole agent, while the others used it in combination with other sedatives or opioids. Although the definitions of recovery time were slightly different across studies, the recovery time could be roughly considered to be the time interval from the completion of the procedure to the achievement of discharge criteria. The pattern of relative time reduction by propofol was observed consistently and clearly when propofol was used alone (weighted mean difference, WMD = −10.38 [−14.34, −6.41], I^2^ = 90.7%). Although the statistical heterogeneity was still large within this subgroup, it was due to the magnitude of the effect, not its direction. When propofol was combined with opioids or when propofol and the control sedative were used in combination with the same concomitant drug, the heterogeneity became greater in terms of the magnitude of the effect, but with a clear tendency for reduction in time. However, when propofol in combination with another sedative was compared with another sole sedative, the results showed a different trend. An overall pooled effect size was therefore not generated.

### Haemodynamic responses

An overall tendency was observed for heart rate (HR) to increase and for mean blood pressure (MBP) to decrease in response to propofol use alone or in combination with another sedative or opioid, although considerable statistical heterogeneity was also observed (Supplementary Fig. S3).

### Minor adverse events

Two RCTs reported data on coughing (Supplementary Fig. S4). In a small RCT, incidence of coughing was observed in 7 of 10 children in the propofol group, while none was observed in 20 children from the midazolam or the ketamine groups (risk difference, RD = 0.80 [0.43, 1.00] and 0.60 [0.19, 1.00]) respectively. However, there was no statistically significant difference between patients who received propofol combined with an opioid and those who received combinations of other sedatives (RD = −0.02 [−0.05, 0.02]). These results were not pooled together due to heterogeneity.

There was no significant overall difference in the incidence of nausea or vomiting between regimens that used propofol and the comparator groups (RD = −0.02 [−0.06, 0.02], I^2^ = 46.4%) from 10 RCTs with 12 comparisons (Supplementary Fig. S4). Propofol was associated with a significant reduction in the incidence of nausea or vomiting in some those studies^33,37^, but these findings were not clearly characterized. One study^12^ had a substantially higher rate of nausea and vomiting than other studies, but did not cause statistically significant heterogeneity in terms of treatment difference.

Nine studies evaluated the occurrence of emergence agitation at various time points. A marginally significant risk reduction was observed overall when propofol was used (RD = −0.03 [−0.06, 0.01], I^2^ = 55.3%) (Supplementary Fig. S4).

### Moderate adverse events

Eighteen studies with 19 comparisons reported cardiovascular problems. The risk of hypotension did not differ between regimens that used propofol and other sedative or opioid groups (RD = 0.00 [−0.01, 0.01], I^2^ = 3.8%) in 15 studies. Bradycardia and tachycardia differed only minimally between the regimens that used propofol and other sedative groups, without statistical significance (RD = 0.01 [−0.02, 0.03], I^2^ = 34.1% from 10 studies for bradycardia; RD = −0.02 [−0.07, 0.03], I^2^ = 0.0% from 3 studies with 4 comparisons for tachycardia) (Supplementary Fig. S5).

Twenty-two studies with 27 comparisons provided information on respiratory complications, and there was an overall trend towards an increased risk of respiratory adverse events when propofol was used (Supplementary Fig. S6). However, the tendency of increasing risk was not statistically significant in terms of the incidence of a decreased respiratory rate (RD = 0.03 [−0.01, 0.08], I^2^ = 56.4% from 8 studies with 10 comparisons). Although there was a significant difference in the incidence of oxygen desaturation between the regimens using propofol and comparator groups, with an exaggerated observation in a small study^35^, the RD and 95% CI were in fact small (RD = 0.04 [0.01, 0.06], I^2^ = 37.4% from 19 studies). Incidence of hypercapnia was reported in only 2 studies with 5 comparisons. A higher incidence of hypercapnia was found in the patients who received only propofol (13/25) compared with the other 4 comparators based on midazolam (0/25) or ketamine (0/26) as mono-sedatives, in combination (2/25), or administered jointly with an opioid (7/25) in 1 study^38^, while in the other study, no significant difference was observed between patients who received propofol and midazolam and those who received dexmedetomidine with midazolam (1/22 vs 2/22)^15^. An overall estimate for this outcome was not calculated.

### Major adverse events

**Figure 2:**
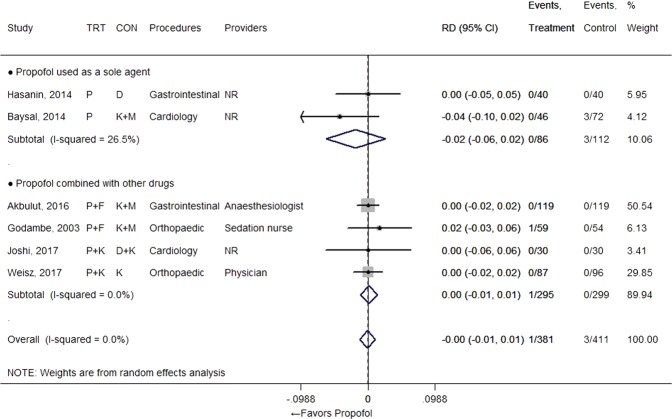
Forest plot for laryngospasm. Abbreviations: CI, confidence interval; CON, control; D, dexmedetomidine; F, fentanyl; K, ketamine; M, midazolam; NR, not reported; P, propofol; RD, risk difference; TRT, treatment.

**Figure 3:**
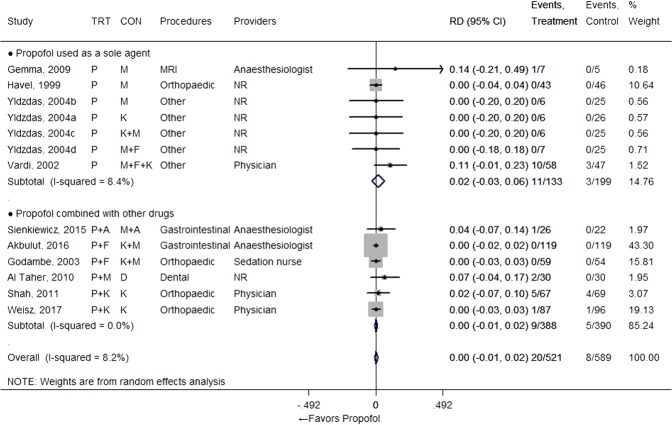
Forest plot for apnoea. Abbreviations: A, alfentanil; CI, confidence interval; CON, control; D, dexmedetomidine; F, fentanyl; K, ketamine; M, midazolam; MRI, magnetic resonance imaging; NR, not reported; P, propofol; RD, risk difference; TRT, treatment.

**Figure 4:**
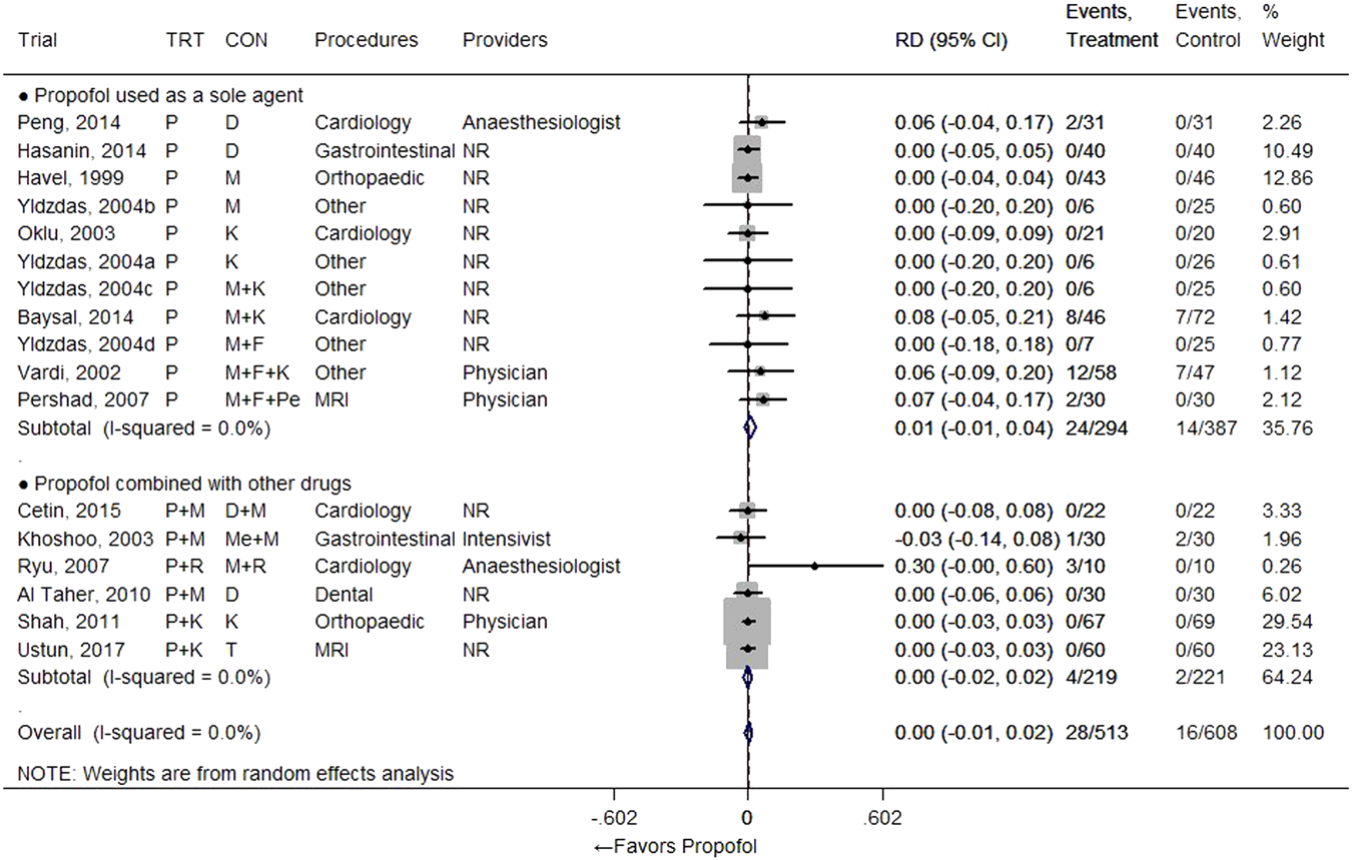
Forest plot for need for airway support. Abbreviations: CI, confidence interval; CON, control; D, dexmedetomidine; F, fentanyl; K, ketamine; M, midazolam; MRI, magnetic resonance imaging; Me, Meperidine; Mt, methohexital; NR, not reported; P, propofol; Pe, pentobarbital; R, remifentanil; RD, risk difference; T, Thiopental; TRT, treatment.

Both laryngospasm and apnoea occurred rarely (in fewer than 5% of patients), whether they received propofol or not (Figs 2 and 3). Therefore, there was no significant RD observed for those events between regimens that used propofol and the comparator groups (RD = 0.00 [−0.01, 0.01], I^2^ = 0.0% from 6 studies for laryngospasm; 0.01 [−0.01, 0.02], I^2^ = 8.2% from 10 studies with 13 comparisons for apnoea). One study presented a relatively high apnoea percentage compared to other studies, but the resulting RD was still small even when this result was included^19^. Need for airway support was also observed rarely in many studies, whether propofol was used or not, with no RD between groups (RD = 0.00 [−0.01, 0.02], I^2^ = 0% from 14 studies with 17 comparisons) (Fig. 4). One study also reported the need for airway support, but we did not include those results in the meta-analysis since its dramatically different scale of incidence (30/88 vs 47/87) was not actually related with the sedation regimen; the event was more broadly defined in the study^36^.

### Investigation of small study effects and publication bias

Funnel plot asymmetries were investigated for the outcomes of bradycardia, need for airway support, hypotension, reduced respiratory rate, desaturation, and apnoea. The contour-enhanced funnel plots were not observed to be asymmetrical, and the *P* values for the Egger test were 0.995 and 0.435 for the incidence of bradycardia and the incidence of need for airway support, respectively (Supplementary Figs S8 and S12). However, statistically significant small study effects were observed for all other adverse events (*P* value for the Egger test: 0.036, 0.043, 0.011, and 0.029, respectively). Some small studies that used inadequate or unclear methods of randomisation and blinding^19,21,25,35^ were in fact found to report a significantly higher incidence of those adverse events in the groups using propofol than in the comparator groups (Supplementary Figs S7, S9–S11). Visual inspection of the contour-enhanced funnel plots implies that some counterpart small studies might have been missing. After adding the potentially missing studies by using the trim and fill method, the pooled RD in those adverse events shifted towards zero. In particular, the resulting RD in the incidence of desaturation decreased from 0.04 (95% CI = [0.01, 0.06]) to 0.02 (95% CI = [−0.01, 0.04]) and was no longer significant.

## Discussion

We sought to evaluate the overall safety of using propofol for procedural sedation in paediatric patients covering a broad range of clinical settings. The terminology for propofol sedation or anaesthesia is often confusingly used because sedation is a continuum and children can easily slip into a deeper level^4^. However, generally, a distinction between planned sedation and planned general anaesthesia is made depending on whether an invasive airway device such as a laryngeal mask airway or endotracheal tube is needed^42^. Therefore, when the use of an invasive airway device was planned, we classified the intended level of sedation as general anaesthesia. In addition, we excluded studies where propofol was used as an adjunct to another anaesthetic agent, or where propofol was used for maintenance of anaesthesia after induction.

Our meta-analysis included 30 studies incorporating 3,774 children who received propofol, other sedative agents, or opioids for a variety of procedures. We found that numerous treatment strategies were adopted for paediatric sedation during non-painful or distressing procedures. For non-painful procedures, propofol alone was used in most studies. For painful procedures, propofol in combination with opioids was used in many studies. In contrast, the treatment regimens of the control drugs were much more diverse. Although the dose of propofol in the combination regimen with other drugs would be less than in the propofol-only sedation regimen, we found that the incidence of some dose-dependent side effects, such as hypotension and reduced respiratory rate, was generally similar across studies. In some small studies, the rate of hypotension, reduced respiratory rate, desaturation, and apnoea tended to be higher, which generated asymmetric skewed funnel plots. Further analysis using the trim and fill method suggested that the use of propofol sedation likely had no significant associations with the higher occurrence of respiratory and cardiovascular adverse events.

A previous large multi-institutional observational study without controls investigated adverse events during paediatric sedation with propofol for procedures^4^. This study, carried out by the Paediatric Sedation Research Consortium, suggested that propofol sedation is unlikely to have serious adverse outcomes, such as mortality and cardiac arrest, and the authors also noted that such results rely on the ability of institutions to manage less serious events, including laryngospasm, airway obstruction, and apnoea. Our study focused on potentially serious or moderate adverse events associated with propofol, in comparison with other sedative or opioid drugs, and our findings suggest that the incidence of those adverse events in the propofol regimen groups, using propofol either as a sole agent or in combination with other sedatives or opioids, was similar to the incidence in the control groups. The previous study showed that adverse events were significantly associated with patients’ American Society of Anesthesiologists status and age group, but such an investigation was not feasible in our study due to the nature of the analysis based on aggregated data extracted from published studies. Although we also tried to provide information on the type of provider along with results of the analysis to explore the impact of this factor on the safety results, no formal analysis could be performed since the relevant information was not available in about 43% of studies included in the analysis. However, based on the descriptive information that we extracted and presented along with the results of safety outcomes, no notable trend associated with these factors was observed.

We acknowledge that this study has several limitations. First, since this meta-analysis was conducted based on RCTs, it is possible that the trials may have been conducted in ideal circumstances by better qualified and experienced physicians than is likely to be the case in usual clinical settings. This factor may have influenced the trial results; for example, the incidence of adverse events might have been underestimated. However, this limitation would apply to both the propofol and control groups, and assessing the comparative differences is still expected to yield meaningful results. Second, although we attempted to extract data on the dosage of the agents involved, it was in fact very complicated to summarize dosing in a single framework, since dosing is related to several other factors, including the type of procedures, level of sedation, and combination of drugs. When some studies showed particularly different safety outcome results, we therefore tried to explain qualitatively how those studies differed from others in terms of the use of the agents. When some studies presented substantially higher incidence rates in both groups, causing statistically significant heterogeneity, for reasons that were not related to the use of the agents, we described them qualitatively, but excluded them from the meta-analysis. Third, although only a small number of studies were considered to have a high risk of bias based on the quality assessment, several studies with an unclear risk of bias due to a lack of information regarding methods of randomisation or blinding were included in the meta-analysis. We endeavoured to explain the potential impact of study quality in relation to the results of specific outcomes.

In conclusion, propofol sedation had advantages in recovery time compared with other drugs, without excessive concerns for cardiovascular or respiratory adverse events. Its safety profile regarding coughing, nausea or vomiting, and emergence delirium was also similar to that of other drugs. Taken together, the overall evidence suggests that propofol could be considered for sedation for paediatric procedures as an option that is comparable to other alternatives. The use of propofol sedation for paediatric procedures should be considered for regulatory approval.

## Methods

### Search methods for identification of studies

We carried out a search for published articles using MEDLINE, Embase, and the Cochrane Central Register of Controlled Trials from their inception to January 31, 2018. The main keywords used for the search were *infant, child, adolescent, propofol, sedation*, and *randomised controlled trial* (Supplementary Table S2).

### Selection criteria

Studies were considered eligible if they met the following criteria: (1) including children under the age of 19; (2) comparing propofol or propofol combination regimens with other sedative agents or opioids for procedural sedation, (3) providing data from safety assessments, and (4) employing a parallel RCT design. Papers in English were considered. Letters to the editor, abstracts, and proceedings of meetings were considered only for identifying relevant studies.

### Data extraction

The following items were extracted from each article: the first author’s name; publication year; participants’ age; number of randomised patients; the type of procedure undertaken; the type of provider; American Society of Anesthesiologists class; details of the intervention and control treatments; and outcome results including recovery time, haemodynamic responses, and adverse events. The adverse events were classified as minor, moderate, or major by the severity or clinical importance of events according to the adverse event reporting tool^4,43,44^. When haemodynamic responses were measured repeatedly during drug infusion, the minimum values of the haemodynamic responses (HR, MBP) were extracted. When studies had multiple treatment arms, each pairwise comparison with a ‘shared’ group was included in the meta-analysis, and the ‘shared’ group was divided out evenly among the comparisons.

### Assessment of the risk of bias in the included studies

We assessed the risk of bias in each of the following domains: adequacy of sequence generation, adequacy of allocation concealment, adequacy of blinding, completeness of data, selective outcome reporting, and other potential threats to validity. The first 4 domains were graded as ‘adequate’ for low risk, ‘inadequate’ for high risk, and ‘unclear’ for uncertain risk when insufficient information was reported to permit a judgement. When any suggestion was found of selective revealing or the suppression of pre-specified outcomes, we assessed the study as having a risk of selective outcome reporting. In studies reporting results drawn from a preliminary analysis, or a sample size of fewer than 25 children, we considered that other potential threats to validity were present.

### Statistical analysis

The WMD and the RD were calculated for continuous and dichotomous data, respectively. A random-effects model was used to calculate pooled effect estimates with 95% confidence intervals, and to test for differences in effects at the 5% significance level when the pooling was considered reasonable without high statistical heterogeneity. Heterogeneity was evaluated using I^2^ statistics, with I^2^ values ≥ 75% considered to suggest high and considerable heterogeneity^45^. A subgroup analysis was done to explore heterogeneity among studies where possible. When it was not plausible to conduct a formal analysis, we attempted to explain the potential sources for the heterogeneous results qualitatively. Some characteristics considered as potential explanations of heterogeneity included the type of procedures performed, the control drugs, the type of provider, the treatment regimen, the definition of outcomes, and study quality.

The presence of publication bias and small study effects were evaluated by a contour-enhanced funnel plot^46^ and the Egger test^47^. When evidence of small study effects was detected, the trim and fill method was used to obtain estimates of the meta-analysis taking the potential bias into account as a sensitivity analysis^48^. For these explorations, we chose all moderate and major adverse events that were reported in a sufficient number of studies.

We used STATA version 12 (Stata Corp., College Station, TX, USA) for all analyses.

### Ethical approval

Institutional Review Board approval was not required because this meta-analysis does not involve human subjects.

## Supplementary information

Supplementary Online Content

## Data Availability

This review uses data and findings from already published studies that are publicly available.
