# Supplementary material for: Evaluation of the safety of using propofol for paediatric procedural sedation: A systematic review and meta-analysis
Source: Sci Rep. 2019 Aug 22;9:12245. doi: 10.1038/s41598-019-48724-x (PMC6706375; doi:10.1038/s41598-019-48724-x)
Supplement: Supplementary file 1 — Supplementary Online Content [file 41598_2019_48724_MOESM1_ESM.docx]

**Evaluation of the safety of using propofol for paediatric procedural sedation: A systematic review and meta-analysis**

Sunhee Kim^1^, Seokyung Hahn^2,3*^, Myoung-jin Jang^3^, Yunhee Choi^3^, Hyunsook Hong^3^, Ji-Hyun Lee^4^, and Hee-Soo Kim^4^

^1^Interdisciplinary Program in Medical Informatics, Seoul National University College of Medicine, Seoul, 03080, Korea;

^2^Department of Medicine, Seoul National University College of Medicine, Seoul, 03080, Korea

^3^Division of Medical Statistics, Medical Research Collaborating Center, Seoul National University Hospital, Seoul, 03080, Korea;

^4^Department of Anesthesiology and Pain Medicine, Seoul National University Hospital, Seoul, 03080, Korea;

^*^ Correspondence to Prof. Seokyung Hahn [hahns@snu.ac.kr](mailto:hahns@snu.ac.kr)

**Figure S1** Trial selection process.

**Table S1.** Characteristics of the 30 randomised controlled trials included in the meta-analysis

**Figure S2** Risk of bias graph of the 30 studies included in the meta-analysis.

**Figure S3** (A) Forest plot for heart rate (B) Forest plot for mean blood pressure.

**Figure S4** (A) Forest plot for coughing (B) Forest plot for nausea or vomiting (C) Forest plot for emergency agitation.

**Figure S5** (A) Forest plot for hypotension (B) Forest plot for bradycardia (C) Forest plot for tachycardia.

**Figure S6** (A) Forest plot for reduced respiratory rate (B) Forest plot for desaturation (C) Forest plot for hypercapnia.

**Figure S7** Investigation of small study effects and publication bias: hypotension (A) Funnel plot **(**B) Egger test for small study effects (C) Trim-and-fill method.

**Figure S8** Investigation of small study effect and publication bias: bradycardia (A) Funnel plot **(**B) Egger test for small study effects.

**Figure S9** Investigation of small study effects and publication bias: reduced respiratory rate (A) Funnel plot **(**B) Egger test for small study effects (C) Trim-and-fill method.

**Figure S10** Investigation of small study effects and publication bias: desaturation (A) Funnel plot **(**B) Egger test for small study effects (C) Trim-and-fill method.

**Figure S11** Investigation of small study effects and publication bias: apnoea (A) Funnel plot **(**B) Egger test for small study effects (C) Trim-and-fill method.

**Figure S12** Investigation of small study effects and publication bias: need for airway support (A) Funnel plot **(**B) Egger test for small study effects.

**Table S2.** Search strategies for MEDLINE via Ovid, Embase, and CENTRAL

**Figure S1** Trial selection process.


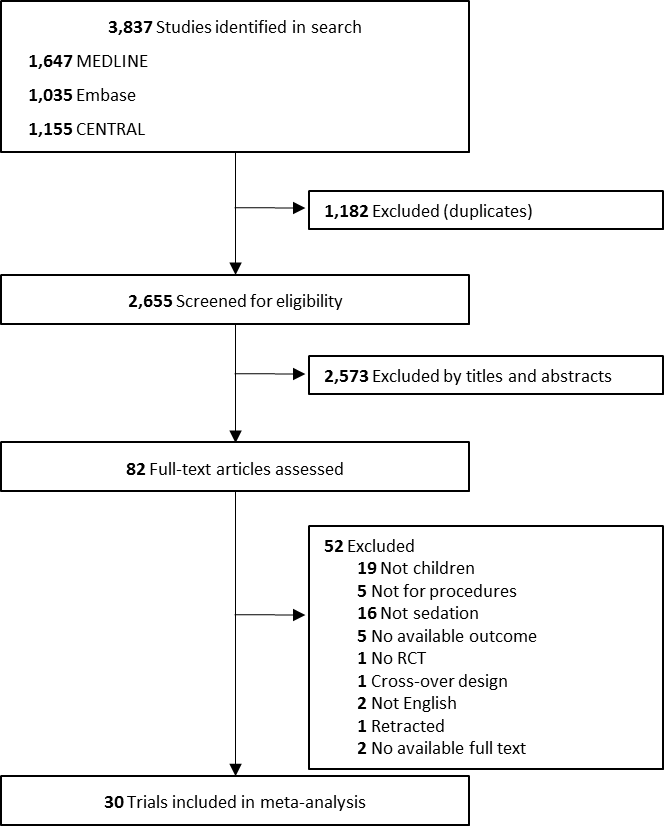


Abbreviations: RCT, randomised controlled trial.

**Table S1.** Characteristics of the 30 randomized controlled trials included in the meta-analysis

| **Source** | **Age ^a^** | **ASA Class** | **Type of Procedures** | **Intervention** | | **No. of Patients** | **Type of Provider** |
| --- | --- | --- | --- | --- | --- | --- | --- |
|  |  |  |  | **Treatment Group** | **Control Group** |  |  |
| Koroglu, 2006 ^14^ | 3.5 (1-7) | I, II | MRI procedures | Propofol | Dexmedetomidine | 60 | Anaesthesiologists |
| Kamal, 2017 ^41^ | 4.7 (2-10) | I, II | MRI procedures | Propofol | Dexmedetomidine | 60 | NR |
| Gemma, 2009 ^29^ | 4.7 (3-7) | I | MRI procedures | Propofol | Midazolam | 12 | Anaesthesiologists |
| Pershad, 2007 ^40^ | 5.6 (1-17) | II, III | MRI procedures | Propofol | Pentobarbital + midazolam + fentanyl | 60 | Sedating physician |
| Kain, 1994 ^13^ | NR (0.9-6.5) | I, II | MRI procedures | Propofol | Pentobarbital + thiopental | 58 | Anaesthesiologists |
| Ustun, 2017 ^34^ | 4.4 (1-12) | I, II | MRI procedures | Propofol + ketamine | Thiopental | 120 | NR |
| Peng, 2014 ^32^ | 10.8 (6-15) | I, II | Cardiology procedures | Propofol | Dexmedetomidine | 62 | Anaesthesiologists |
| Oklu, 2003 ^26^ | 4.5 (0.1-13) | II, III | Cardiology procedures | Propofol | Ketamine | 41 | NR |
| Baysal, 2014^18^ | 6.4 (0.5-16) | II, III | Cardiology procedures | Propofol | Ketamine + midazolam | 118 | NR |
| Joshi, 2017 ^16^ | 5.0 (0.1-10) | NR | Cardiology procedures | Propofol + ketamine | Dexmedetomidine + ketamine | 60 | NR |
| Cetin, 2015 ^15^ | NR (1-18) | I, II | Cardiology procedures | Propofol + midazolam | Dexmedetomidine + midazolam | 44 | NR |
| Ryu, 2007 ^35^ | 5.3 (2-10) | NR | Cardiology procedures | Propofol + remifentanil | Midazolam + remifentanil | 20 | Anaesthesiologists |
| Rai, 2007 ^27^ | NR (3-6) | I, II | Dental procedures | Propofol | Ketamine or midazolam | 30 | Anaesthesiologists |
| Canpolat, 2017 ^33^ | 5.4 (2-8) | I, II | Dental procedures | Propofol + ketamine | Dexmedetomidine + ketamine | 60 | NR |
| Al Taher, 2010 ^20^ | 7.0 (4-10) | I | Dental procedures | Propofol + midazolam | Dexmedetomidine | 60 | NR |
| Canpolat, 2016 ^37^ | 5.6 (3-9) | I, II | Dental procedures | Propofol or propofol + ketamine | Ketamine | 60 | Anaesthesiologists |
| Hasanin, 2014 ^21^ | 9.2 (1-14) | I, II | Gastrointestinal procedures | Propofol | Dexmedetomidine | 80 | NR |
| Sienkiewicz, 2015 ^23^ | 13.1 (9-16) | I, II | Gastrointestinal procedures | Propofol + alfentanil | Midazolam + alfentanil | 51 | Anaesthesiologists |
| Akbulut, 2016 ^22^ | NR (4-17) | I, II | Gastrointestinal procedures | Propofol + fentanyl | Midazolam + ketamine | 238 | Anaesthesiologists |
| Khoshoo, 2003 ^30^ | 8.1 (0.2-18) | NR | Gastrointestinal procedures | Propofol + midazolam | Meperidine + midazolam | 60 | Intensivist |
| Van der Lee, 2016 ^24^ | Preterm | NR | Intubation procedures | Propofol | Morphine + vecuronium | 26 | NR |
| Penido, 2011 ^31^ | Preterm | NR | Intubation procedures | Propofol + remifentanil | Midazolam + remifentanil | 20 | NR |
| Havel, 1999 ^12^ | 8.8 (2-18) | I-III | Orthopaedic procedures | Propofol | Midazolam | 89 | NR |
| Godambe, 2003 ^39^ | 9.2 (3-16) | I, II | Orthopaedic procedures | Propofol + fentanyl | Ketamine + midazolam | 113 | Sedation nurse |
| Shah, 2011 ^17^ | 11 (2-17) | I, II | Orthopaedic procedures | Propofol + ketamine | Ketamine | 136 | Physician |
| Weisz, 2017 ^28^ | 8.8 (3-21) | I, II | Orthopaedic procedures | Propofol + ketamine | Ketamine | 183 | Physician |
| Canpolat, 2012 ^25^ | 2.4 (0.7-5) | I, II | Others (burn wound care) | Propofol + ketamine | Dexmedetomidine + ketamine | 60 | Anaesthesiologists |
| Yldzdas, 2004 ^38^ | NR (2-17) | NR | Others (minor procedures in paediatric intensive care unit) | Propofol | Ketamine or midazolam, or ketamine + midazolam, or midazolam + fentanyl | 126 | NR |
| Bauman, 2002 ^36^ | NR (0.2-12) | NR | Others (other painful procedures) | Propofol + fentanyl | Methohexital + remifentanil | 175 | Physician–nurse team |
| Vardi, 2002 ^19^ | 7.1 (10.1-28) | II, III | Others (paediatric critical care) | Propofol | Ketamine + midazolam + fentanyl | 105 | Physician |
| Abbreviations: ASA, American Society of Anesthesiologists; MRI, magnetic resonance imaging; NR, not reported.  ^a^ Mean (range), years | | | | | | | |

**Figure S2** Risk of bias graph of the 30 studies included in the meta-analysis.


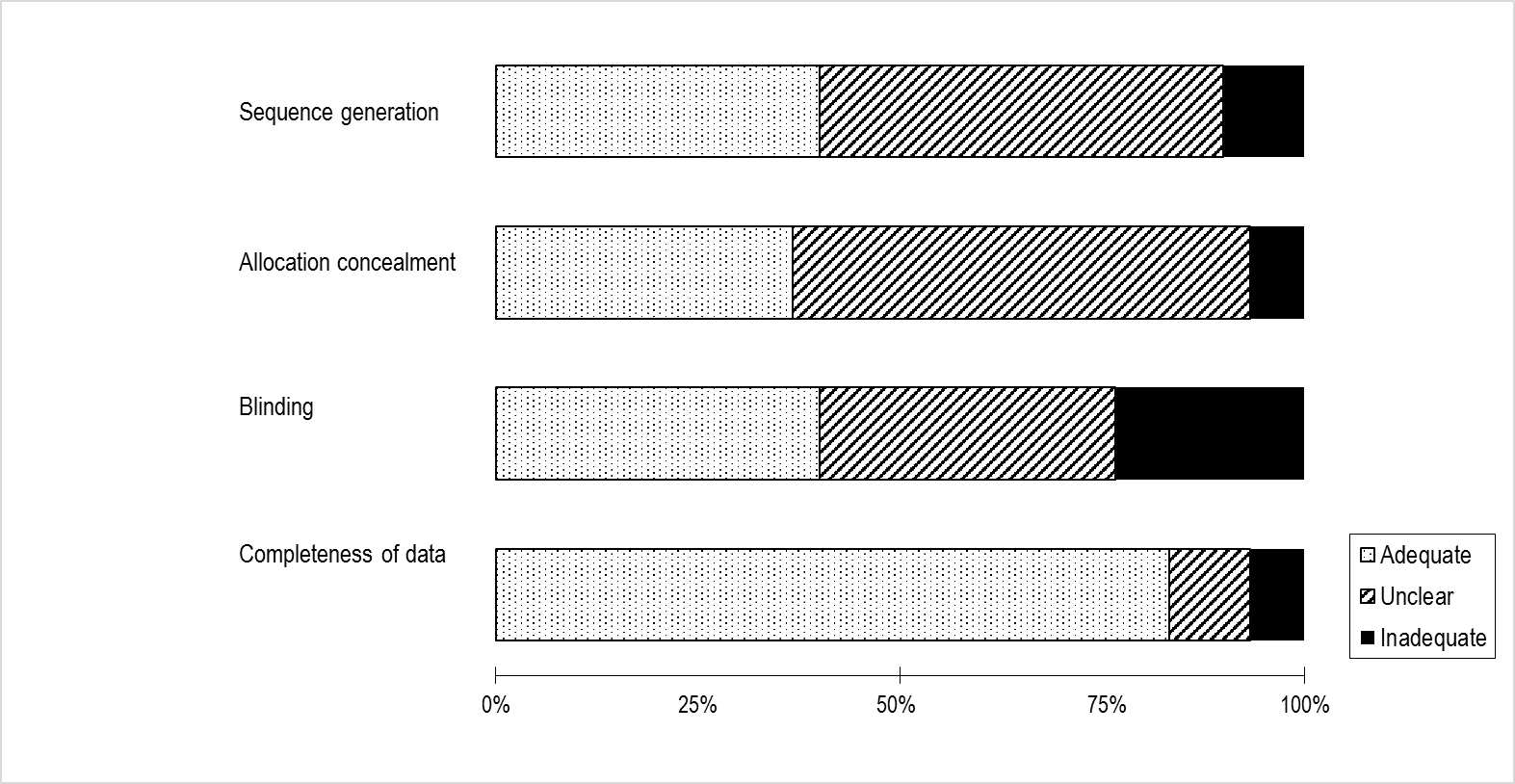


**Figure S3** (A) Forest plot for heart rate

(B) Forest plot for mean blood pressure

Abbreviations: CI, confidence interval; CON, control; D, dexmedetomidine; K, ketamine; M, midazolam; MRI, magnetic resonance imaging; P, propofol; R, remifentanil; SD, standard deviation; TRT, treatment; WMD, weighted mean difference.

**Figure S4**

(A) Forest plot for coughing

(B) Forest plot for nausea or vomiting

(C) Forest plot for emergency agitation

Abbreviations: A, alfentanil; CI, confidence interval; CON, control; D, dexmedetomidine; F, fentanyl; K, ketamine; M, midazolam; MRI, magnetic resonance imaging; Mt, methohexital; NR, not reported; P, propofol; Pe, pentobarbital; R, remifentanil; RD, risk difference; TRT, treatment.

**Figure S5**

(A) Forest plot for hypotension

(B) Forest plot for bradycardia

(C) Forest plot for tachycardia

Abbreviations: CI, confidence interval; CON, control; D, dexmedetomidine; F, fentanyl; K, ketamine; M, midazolam; MRI, magnetic resonance imaging; Mo+V, morphine + vecuronium; Mt, methohexital; NR, not reported; P, propofol; Pe, pentobarbital; R, remifentanil; RD, risk difference; TRT, treatment.

**Figure S6**

(A) Forest plot for reduced respiratory rate

(B) Forest plot for desaturation

(C) Forest plot for hypercapnia

Abbreviations: A, alfentanil; CI, confidence interval; CON, control; D, dexmedetomidine; F, fentanyl; K, ketamine; M, midazolam; Me, Meperidine; NR, not reported; P, propofol; Pe, pentobarbital; R, remifentanil; RD, risk difference; TRT, treatment.

**Figure S7** Investigation of small study effects and publication bias: hypotension

(A) Funnel plot

**(**B) Egger test for small study effects: P-value = 0.036

(C) Trim-and-fill method: RD = 0.00 (95% CI, −0.01, 0.01)

**Figure S8** Investigation of small study effects and publication bias: bradycardia

(A) Funnel plot

**(**B) Egger test for small study effects: P-value = 0.995

**Figure S9** Investigation of small study effects and publication bias: reduced respiratory rate

(A) Funnel plot

**(**B) Egger test for small study effects: P-value = 0.043

(C) Trim-and-fill method: RD = 0.02 (95% CI, −0.01, 0.05)

**Figure S10** Investigation of small study effects and publication bias: desaturation

(A) Funnel plot

**(**B) Egger test for small study effects: P-value = 0.011

(C) Trim-and-fill method: RD = 0.02 (95% CI, −0.01, 0.04)

**Figure S11** Investigation of small study effects and publication bias: apnoea

(A) Funnel plot

**(**B) Egger test for small study effects: P-value = 0.029

(C) Trim-and-fill method: RD = 0.00 (95% CI, −0.01, 0.01)

**Figure S12** Investigation of small study effects and publication bias: need for airway support

(A) Funnel plot

**(**B) Egger test for small study effects: P-value = 0.435

**Table S2. Search strategies for MEDLINE via Ovid, Embase, and CENTRAL**

**MDEDLINE via Ovid search strategy: 1,647 results**

#1. exp Infant/

#2. (newborn* or neonat* or infant* or infancy or baby or babies or toddler* or p?ediatric*).mp.

#3. 1 or 2

#4. exp Child/

#5. exp Pediatrics/

#6. (p?ediatric* or child* or kindergar* or preschool* or kid or kids or schoolchild* or school age or schoolage or girl? or boy? or preteen* or youth* or prepubescent*).mp

#7. 4 or 5 or 6

#8. exp Adolescent/

#9. (adolesc* or teen* or youth* or underage* or ‘under age*’ or minor* or juvenile* or pubert* or pubescen* or young people or young person* or young adult*).mp.

#10. 8 or 9

#11. 3 or 7 or 10

#12. exp Propofol/

#13.(diprivan or propofol or recofol or disoprivan or diprofol or disoprofol or diisoprophylphenol or propofolum or propovan or fresofol or rapinovet or pofol or aquafol or ivofol or "ici-35,868" or "ici35,868" or "ici35,868" or "ici-35868" or "ici35868" or"ici35868").mp.

#14. 12 or 13

#15. randomized controlled trial.pt.

#16. controlled clinical trial.pt.

#17. random*.ab.

#18. trial*.ab.

#19. 15 or 16 or 17 or 18

#20. humans.sh.

#21. 19 and 20

#22. comment.pt.

#23. editorial.pt.

#24. letter.pt.

#25. 22 or 23 or 24

#26. 21 not 25

#27. exp Conscious Sedation/

#28. exp Deep Sedation/

#29. exp "Hypnotics and Sedatives"/

#30. exp Anti-Anxiety Agents/

#31. exp Preanesthetic medication/

#32. (sedat* or non?anaesthetist* or preanesthetic medication or preanaesthetic medication).mp.

#33. (anxiety or anxious or fear* or fright* or stress* or distress* or phobi*).mp.

#34. 27 or 28 or 29 or 30 or 31 or 32 or 33

#35. 11 and 14 and 26 and 34

**Embase search strategy: 1,035 results**

#1. ‘Infant'/exp

#2. (newborn* or neonat* or infant* or infancy or baby or babies or toddler* or paediatric* or pediatric*):ab,ti

#3. #1 or #2

#4. 'child'/exp

#5.'Pediatrics'/exp

#6. (paediatric*or pediatric* or child* or kindergar* or preschool* or kid or kids or schoolchild* or 'school age' or schoolage or girl* or boy* or preteen* or youth* or prepubescent*):ab,ti

#7. #4 or #5 or #6

#8. 'adolescent'/exp

#9.'adolescence'/syn or (adolesc* or teen* or youth* or underage* or “under age*” or minor* or juvenile* or pubert* or pubescen* or (young NEAR/3 people) or (young NEAR/3 person*) or (young NEAR/3 adult*)):ab,ti

#10. #8 or #9

#11. (#3 or #7 or #10) and [embase]/lim

#12. 'Propofol'/exp

#13. (diprivan or propofol or recofol or disoprivan or diprofol or disoprofol or diisoprophylphenol or propofolum or propovan or fresofol or rapinovet or pofol or aquafol or ivofol or "ici-35,868" or "ici35,868" or "ici35,868" or "ici-35868" or "ici35868" or "ici35868"):ab,ti

#14. (#12 or #13) and [embase]/lim

#15. 'randomized controlled trial'/exp

#16. 'controlled clinical trial'/exp

#17. 'randomization'/exp

#18. 'double blind procedure'/exp

#19. 'single blind procedure'/exp

#20. random*:ab

#21. trial*:ab

#22. #15 or #16 or #17 or #18 or #19 or #20 or #21

#23. 'human'/exp

#24. #22 AND #23

#25. editorial:it

#26. letter:it

#27. #25 or #26

#28. (#24 NOT #27) and [embase]/lim

#29. ‘conscious sedation’/exp

#30. ‘deep sedation’/exp

#31. 'hypnotic sedative agent'/exp

#32. ‘sedation’/exp

#33. 'anxiolytic agent'/exp

#34. (sedat* or "non-anaesthetist*" or nonanaesthetist* or “preanesthetic medication” or “preanaesthetic medication”):ab,ti

#35. (anxiety or anxious or fear* or fright* or stress* or distress* or phobi*):ab,ti

#36. (#29 or #30 or #31 or #32 or #33 or #34 or #35) and [embase]/lim

#37. #11 and #14 and #28 and #36

**CENTRAL, The Cochrane Library search strategy: 1,155 results**

#1. MeSH descriptor: [Infant] explode all trees

#2. (newborn*or neonat* or infant* or infancy or baby or babies or toddler* or paediatric* or pediatric*):ti,ab,kw

#3. #1 or #2

#4. MeSH descriptor: [Child] explode all trees

#5. MeSH descriptor: [Pediatrics] explode all trees

#6. (paediatric*or pediatric* or child* or kindergar* or preschool* or kid or kids or schoolchild* or school age or schoolage or girl* or boy* or preteen* or youth* or prepubescent*):ti,ab,kw

#7. #4 or #5 or #6

#8. MeSH descriptor: [Adolescent] explode all trees

#9. (adolesc*or teen* or youth* or underage* or ‘under age*’ or minor* or juvenile* or pubert* or pubescen* or young people or young person* or young adult*):ti,ab,kw

#10. #8 or #9

#11. #3 or #7 or #10

#12. MeSH descriptor: [Propofol] explode all trees

#13. (diprivan or propofol or recofol or disoprivan or diprofol or disoprofol or diisoprophylphenol or propofolum or propovan or fresofol or rapinovet or pofol or aquafol or ivofol or "ici-35,868" or "ici35,868" or "ici35,868" or "ici-35868" or "ici35868" or "ici35868"):ti,ab,kw

#14. #12 or #13

#15. MeSH descriptor: [Conscious Sedation] explode all trees

#16. MeSH descriptor: [Deep Sedation] explode all trees

#17. MeSH descriptor: [Hypnotics and Sedatives] explode all trees

#18. MeSH descriptor: [Anti-Anxiety Agents] explode all trees

#19. MeSH descriptor: [Preanesthetic Medication] explode all trees

#20. (sedat* or non?anaesthetist* or preanesthetic medication or preanaesthetic medication):ti,ab,kw

#21. (anxiety or anxious or fear* or fright* or stress* or distress* or phobi*):ti,ab,kw

#22. #15 or #16 or #17 or #18 or #19 or #20 or #21

#23. #11 and #14 and #22
